# O071: Standard precautions (SP) vs ‘search-and-destroy’ strategy for control of methicillin-resistant Staphylococcus aureus (MRSA) in nursing homes (NH): a randomized controlled study

**DOI:** 10.1186/2047-2994-2-S1-O71

**Published:** 2013-06-20

**Authors:** C Bellini, C Petignat, E Masserey, C Büla, B Burnand, D Blanc, G Zanetti

**Affiliations:** 1 University Hospital CHUV, Switzerland; 2 Public Health, Lausanne, Switzerland

## Introduction

MRSA prevalence among NH residents of Canton Vaud, Switzerland, rose from 4.5% in 2003 to 12% in 2008. MRSA control strategy is not clearly defined in this setting.

## Objectives

The aim of our study was to measure the 1-year impact of universal screening followed by decolonization of carriers (intervention), compared with SP (control), on MRSA prevalence in NH.

## Methods

104/157 NH participated to this randomized controlled study. In participating NH, SP were enforced and residents underwent MRSA screening at study entry, upon (re)admission, and at 12 months. All MRSA carriers in intervention NH underwent a 5-day topical decolonization (nasal mupirocine, chlorhexidine disinfection of skin and pharynx) combined with environmental disinfection, except if they had ongoing MRSA infection or bacteriuria or stage IV skin ulcers. Decolonization was repeated once in case of failure.

## Results

A total of 6’036 residents (51% in control and 49% in intervention NH) were screened, representing 86% [27-100%] of the NH population in the control and 87% [20-100%] in the intervention NH. Characteristics of NH (size, single rooms proportion, healthcare workers/resident) and of residents (age, gender, diabetes, ulcers, medicals devices, performance score, previous admission in acute care hospital, previous antibiotics) were similar in both groups. In intervention NH, 209/274 (76%) MRSA carriers underwent decolonization, with success in 61%. At study end, proportions of the initial MRSA carriers who were no longer positive at screening were 65% in intervention NH and 55 % in control NH (p=.07). Nevertheless, the impact of the intervention did not reach significance: mean MRSA prevalence significantly declines from 8.9% in both groups [0- 44%] to 6.6 % in the control group and to 5.8% in the intervention group (p=.3).

## Conclusion

Topical MRSA decolonization was successful in 60% of NH residents, which opens interesting perspectives for high-risk individuals. At NH level, however, universal screening followed by decolonization of carriers had no significant additional impact in reducing prevalence of MRSA carriage rate at one year compared to SP and spontaneous decolonization.

## Disclosure of interest

None declared.

